# Determination and Prediction of the Energy Content and Amino Acid Digestibility of Enzymolytic Soybean Meal for Growing Pigs

**DOI:** 10.3390/ani16040620

**Published:** 2026-02-15

**Authors:** Ya Wang, Chengling Bao, Xiaofeng Guan, Yanchu Yao, Jinxiu Huang

**Affiliations:** Institute of Animal Nutrition, Chongqing Academy of Animal Sciences, Chongqing 402460, China; wangya920708@163.com (Y.W.); baochengling@163.com (C.B.); ggguanxf@163.com (X.G.)

**Keywords:** energy, amino acid, predictive model, enzymolytic soybean meal, growing pigs

## Abstract

This study investigated the nutritional value of enzymolytic soybean meal (**ESBM**) in growing pigs through two experiments. **Experiment 1** evaluated the digestible energy (**DE**) and metabolizable energy (**ME**) values using twenty-two crossbred pigs (Duroc × Landrace × Yorkshire; initial BW 36.47 ± 0.63 kg) in a replicated 11 × 3 incomplete Latin square design. The dietary treatments comprised a corn-based diet and ten test diets formulated by substituting 30% corn with ESBM. **Experiment 2** evaluated standardized ileal digestibility (**SID**) of amino acid (**AA**) through a 10 × 6 incomplete Latin square design involving ten pigs (Duroc × Landrace × Yorkshire; initial BW: 21.30 ± 1.38 kg) fitted with T-cannulas. All experimental diets contained 40% ESBM as the sole source of AA and 0.2% titanium dioxide as an indigestible marker. The results revealed variations in the chemical composition of ESBM samples, with coefficients of variation (**CV**) exceeding 9% for all analyzed components except for gross energy (**GE**), which had a CV of 4.65%. The mean DE and ME were 16.46 MJ/kg DM (13.82 to 19.13 MJ/kg DM) and 15.73 MJ/kg DM (12.79 to 18.77 MJ/kg DM), respectively. The best-fit prediction equations for DE and ME were as follows: DE (MJ/kg DM) = −26.31 + (2.74 × GE) − (0.17 × CP) (R^2^ = 0.76, *p* < 0.01) and ME (MJ/kg DM) = −28.45 + (2.85 × GE) − (0.19 × CP) (R^2^ = 0.70, *p* = 0.01). The mean SID of Lys, Met, Thr, Trp, Val, and total AA were 81.72% (71.19 to 95.64%), 81.36% (45.59 to 95.76%), 76.19% (59.67 to 90.40%), 50.61% (15.40 to 74.13%), 81.23% (69.39 to 92.99%), and 84.29% (67.36 to 96.56%), respectively. In this study, ESBM5 exhibited higher energy content and AA digestibility, whereas ESBM8 demonstrated the lowest nutritional value. The ash content exhibited the strongest negative correlation with DE (r = −0.75; *p* < 0.05) and ME (r = −0.76; *p* < 0.05), while Trp showed the strongest positive correlation with the SID of AA. The chemical composition of ESBM can be effectively used to predict DE, ME, and SID of AA in growing pigs.

## Simple Summary

Enzymolytic soybean meal (**ESBM**) is commonly incorporated into swine diets as a high-quality protein supplement. Nevertheless, considerable variability in chemical composition exists among ESBM samples from different manufacturers, primarily due to differences in soybean meal sources and enzymatic processing technologies. Moreover, limited data have been reported on the nutritional value of ESBM from different sources for growing pigs. Therefore, this study selected ten ESBM samples produced by representative enterprises from eight provinces in China. We systematically compared their chemical composition, energy content, and amino acid digestibility in growing pigs. Results revealed significant variation in digestible energy, metabolizable energy, and amino acid digestibility among the ESBM samples, and regression equations were established based on chemical composition. This study provides valuable references for the precise application of ESBM in pig diet formulations.

## 1. Introduction

Soybean meal (**SBM**), a crucial plant protein source for swine which is the most widely used protein feed ingredient in China, contains 43% to 46% crude protein (**CP**) and exhibits a balanced amino acid (**AA**) profile [1,2]. However, this high-quality byproduct contains allergenic proteins (e.g., glycinin and β-conglycinin) and key anti-nutritional factors (**ANFs**), including trypsin inhibitors and phytic acid [3,4]. These components can impair nutrient digestion, absorption, and intestinal health [5]. Consequently, reducing allergenic proteins and ANFs in SBM through pretreatment technologies is critical for improving its feeding value [6].

Enzymatic hydrolysis technology has been widely used to modify SBM due to its mild reaction conditions, substrate specificity, and capacity to produce bioactive peptides [7]. Exogenous proteases can degrade soybean antigenic proteins, while enzymes such as cellulases and phytases effectively break down fibrous polysaccharides and phytate phosphorus, respectively [8], reducing ANF levels. However, standardized industrial-scale hydrolysis parameters—including enzyme combinations, duration, pH, and temperature—are currently lacking in China. Furthermore, substantial variations exist in SBM sources (e.g., variety, origin, and processing methods) [9], leading to considerable product heterogeneity in commercial enzymolytic soybean meal (**ESBM**). This heterogeneity manifests in nutritional composition, residual ANF content, and the distribution of small peptide molecular weights. While current research primarily focuses on evaluating the feeding effects of ESBM, data on the digestible energy (**DE**), metabolizable energy (**ME**), and standardized ileal digestibility (**SID**) of AA in ESBM from different sources remain scarce.

Therefore, this study selected representative commercial ESBM products from large-scale Chinese manufacturers. We determined the DE, ME, and SID of AA and further developed prediction models based on chemical composition. The findings provide reference data for the application of ESBM in pig diets.

## 2. Materials and Methods

All animal procedures were approved by the Animal Care and Use Committee of the Chongqing Academy of Animal Sciences (Approval No. XKY-20221203).

### 2.1. Enzymolytic Soybean Meal Collection

**Figure 1:**
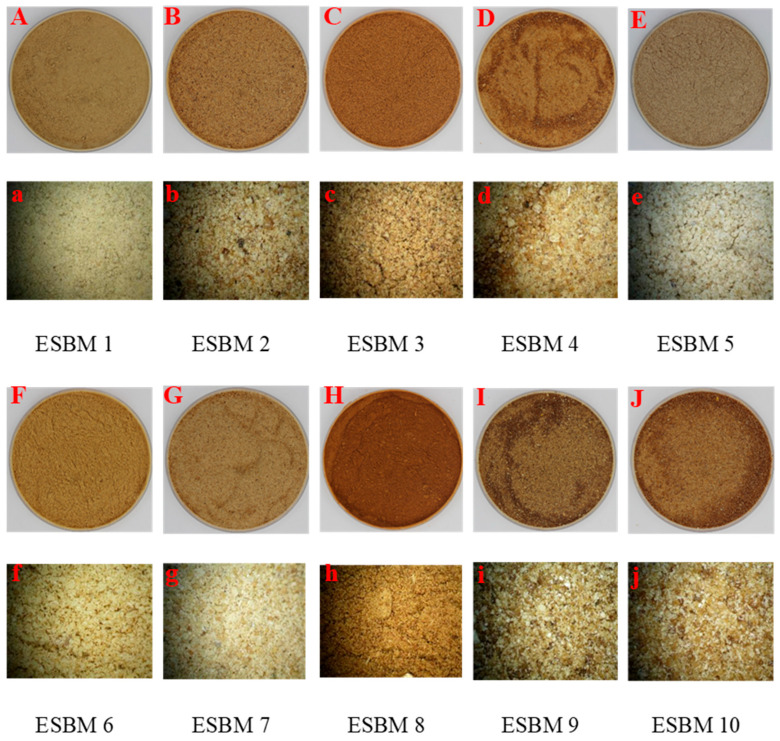
Appearance of the 10 enzymolytic soybean meal (ESBM) samples. These photos were taken with a digital SLR camera (Canon EOS 600D, Tokyo Metropolis, Japan; No. (**A**–**J**)) and a stereomicroscope (Leica M205 FA, Wetzlar, Germany; No. (**a**–**j**), ×50).

Before sampling, investigations were first conducted into the major manufacturers of ESBM in China, and samples were collected for chemical composition analysis. The sampling process was carried out with reference to GB/T 14699.1-2005 [10]. Ten representative ESBM samples were collected from eight major manufacturers across China. The detailed specifications are summarized in Table 1. The morphological characteristics observed under light microscopy are depicted in Figure 1.

### 2.2. Experiment 1: Energy Content Measurements

#### 2.2.1. Animals, Diets, and Experimental Design

A replicated 11 × 3 incomplete Latin square design comprising three consecutive periods was used to determine the DE and ME of ten ESBM samples. Twenty-two crossbred castrated male pigs (DLY), with an initial body weight (**BW**) of 36.47 ± 0.63 kg, were assigned to this design. Dietary treatments included a corn-based diet and 10 test diets, in which 30% corn was replaced by ESBM (Table 2). Six replicates per treatment, with one pig per replicate. Each period consisted of a 7-day adaptation phase followed by a 5-day total collection of feces and urine, with pigs and periods serving as blocking factors. We formulated a basal diet with corn as the sole energy source, vitamin–mineral premixes were supplemented to meet or exceed NRC (2012) requirements for 25 to 50 kg growing pigs [1]. Analyzed dietary compositions are detailed in Table 3. Pigs were housed in metabolism crates (1.8 × 0.7 × 0.9 m, room temperature maintained at 22 ± 2 °C), each fitted with a nipple drinker, feeder, and separate trays for fecal and urinary collection. *Ad libitum* access to water was provided, and the daily feed intake was set at 4% of initial BW [11], divided into two equal meals administered at 0800 and 1500 h, respectively. BW was recorded at the beginning of each period to calculate the feed intake.

#### 2.2.2. Sample Collection and Preparation

Throughout the collection phase, daily feed intake, fecal output, and urinary volume were accurately recorded for each pig. Total collections of urine and feces were conducted for 5 days following the adaptation period [12]. Specifically, urine samples were collected daily in plastic buckets containing 50 mL of 6 N HCl, and total volumes were recorded for each pig. A 10% aliquot of daily urine output was stored at −20 °C for subsequent analysis. The gross energy (**GE**) content of the urine was determined by drying 4 mL samples in a forced-air oven at 65 °C for 8 h [13]. Fecal samples were collected immediately after defecation, sealed in pre-labeled polyethylene bags, and stored at −20 °C. After thawing, a representative 500 g subsample was oven-dried (65 °C, 72 h).

### 2.3. Experiment 2: Amino Acids Digestibility Measurements

#### 2.3.1. Animals, Diets, and Experimental Design

Ten crossbred barrows (DLY; initial BW 21.30 ± 1.38 kg), each equipped with a T-cannula in the distal ileum, were used in a 10 × 6 incomplete Latin square design. The design comprised 10 experimental diets tested over six consecutive periods, with pigs and periods treated as blocking factors (six replicates per diet). Experimental diets were formulated to contain the selected ESBM as the sole source of CP and AA. The detailed formulations of the experimental diets and the analyzed AA profiles are presented in Table 4 and Table 5, respectively. Vitamin–mineral premixes met or exceeded NRC (2012) nutrient requirement for 25 to 50 kg growing pigs [1]. Additionally, titanium dioxide (TiO_2_) was included at 0.20% as an inert marker to estimate the SID of AA.

Feeding protocols were consistent with those detailed in Experiment 1. Each experimental period comprised a 5-day adaptation phase followed by a 2-day ileal digesta collection phase.

#### 2.3.2. Sample Collection and Preparation

Ileal digesta was collected following the standardized protocol [14]. Ileal digesta was collected continuously for 12 h (0800 to 2000 h) into plastic bags attached to the cannula barrel [15]. The bags were removed every 30 min or as soon as they were filled and immediately frozen at −20 °C. After the 2-day collection period, digesta from the same pig within the same period was thawed, pooled, mixed thoroughly, sub-sampled, and then stored at −20 °C until analysis.

### 2.4. Chemical Analysis

In Experiment 1, all ESBM samples, experimental diets, and dried fecal samples were ground through a 0.45 mm sieve. In Experiment 2, ileal digesta and diets were ground through a 0.3 mm sieve. Chemical analyses for both experiments followed the standardized protocols [15]. The Chinese national standards (announced by the General Administration of Quality Supervision, Inspection and Quarantine of the People’s Republic of China and Standardization Administration of China) were used to determine the contents of dry matter (**DM**; GB/T 6435-2014), ether extract (**EE**; GB/T 6433-2006), GE (GB/T 45104-2024), ash (GB/T 6438-2007), CP (GB/T 6432-2018), starch (GB/T 20194-2018), dietary fiber (SDF, IDF, TDF) (GB 5009.88-2023), minerals (K, Na, Mg, Cu, Fe, Mn, Zn) (GB/T 13885-2017),P (GB/T 6437-2018), Ca (GB/T 6436-2018), AA (GB/T 18246-2019); crude fiber (**CF**; GB/T 6434-2022), and neutral detergent fiber (**NDF**; GB/T 20806-2022) [16,17,18,19,20,21,22,23,24,25,26,27,28]. The acid detergent fiber (**ADF)** was determined according to NY/T 1459-2022 [29]. The ANFs were analyzed using the HPLC-MS/MS system. All chemical composition analyses were performed repeatedly.

### 2.5. Calculations

We calculated the DE and ME values for the diets in Experiment 1 using the following calculations [30]:

DE_d_ (MJ/kg DM) = (GE_i_ − GE_f_)/F

ME_d_ (MJ/kg DM) = (GE_i_ − GE_f_ − GE_u_)/F

DE_dc_ (MJ/kg DM) = DE_d_/0.97

ME_dc_ (MJ/kg DM) = ME_d_/0.97

The DE and ME of the ESBM ingredients were calculated using the following calculations:

DE_ESBM_ (MJ/kg DM) = [DE_d_ − DE_dc_ × (100% − X%)]/X%

ME_ESBM_ (MJ/kg DM) = [ME_d_ − ME_dc_ × (100% − X%)]/X%

where DE_d_ and ME_d_ denote the DE and ME of diet; GE_i_ is the total GE intake (diet GE × feed intake F over 5 d); GE_f_ and GE_u_ are the GE content in feces and urine (5 d); DE_dc_ and ME_dc_ correspond to the adjusted DE and ME in the basal diet, with 0.97 representing the proportion of energy-supplying ingredients; DE_ESBM_ and ME_ESBM_ are the DE and ME of each ESBM sample; and X% is the percentage of diet energy supplied by ESBM.

The apparent ileal digestibility (**AID**), SID, and ileal endogenous losses of AA (**IAA**) were calculated using the following calculations [14]:

AID, % = [1 − (AA_1_/AA_2_) × (IM_2_/IM_1_)] × 100;

IAA = AA_3_ × (IM_4_/IM_3_);

SID, % = AID + (IAA/AA_2_) × 100.

where IM_1_ and IM_2_ are TiO_2_ content (mg/kg DM) in digesta and diet, respectively; AA_1_ and AA_2_ are AA content (mg/kg DM) in digesta and diet, respectively; IAA is ileal endogenous loss of AA (mg/kg DM intake). AA_3_ and IM_3_ are AA and TiO_2_ content (mg/kg DM) in ileal digesta from the N-free diet; IM_4_ is TiO_2_ content (mg/kg DM) in the N-free diet.

The IAA values (g/kg DMI) used for the calculation of SID were derived from a previous study conducted by our group [15], which utilized the same breed of pig, a similar body weight rank, and identical surgical and housing conditions.

### 2.6. Statistical Analyses

Statistical analysis was conducted with SAS 9.2 software (SAS Inst. Inc., Carry, NC, USA). Normality was assessed using the UNIVARIATE procedure (NORMAL and PLOT options), and homogeneity of variance was evaluated using Levene’s test across treatments. For data satisfying both assumptions, a mixed-effects model was fitted using PROC MIXED, with the pig as the experimental unit. The statistical model included diet as a fixed effect, and animal and period as random effects. Treatment comparisons were conducted using Tukey’s test with letter-based groupings. Relationships between chemical composition and DE, ME, and SID of AA were analyzed using PROC CORR. Prediction equations for these response variables were developed using PROC REG [31]; model fit was evaluated by maximizing R^2^ and minimizing RMSE. For all analyses, significance and tendencies were defined at *p* < 0.05 and 0.05 ≤ *p* < 0.10, respectively.

## 3. Results

### 3.1. Chemical Composition of ESBM Samples

As shown in Table 6, the CV of GE was 4.65%, while the CVs of CP, EE, CF, NDF, ADF, ash, starch, IDF, SDF, TDF, and AA exceeded 9%. There was considerable variation in the concentrations of minerals and ANFs (Table 7 and Table 8), with the CV ranging from 12.19% to 123.16%. The averaged concentrations of DM, GE, CP, EE, CF, NDF, ADF, and ash in the 10 ESBM samples were 92.48% (88.32 to 95.82%), 17.58 MJ/kg (16.06 to 18.76 MJ/kg), 49.62% (40.29 to 58.60%), 0.96% (0.21 to 3.05%), 5.77% (4.42 to 8.87%), 12.93% (10.07 to 17.72%), 9.49% (7.21 to 13.67%), and 7.12% (3.89 to 9.26%), respectively. The averaged concentrations of Lys, Met, Thr, Trp and Val were 2.84%, 0.37%, 1.84%, 0.56% and 2.33%, respectively. For the two samples from the same manufacturer, ESBM 6 and ESBM 7 showed differences in EE, NDF, and starch; while ESBM 9 and ESBM 10 exhibited differences in CP, EE, CF, NDF, starch, and AA content.

### 3.2. Available Energy in ESBM

As shown in Table 9, on DM basis, there was a significant difference in the DE and ME content (*p* < 0.01). ESBM 5 had the greatest DE and ME, whereas ESBM 8 had the lowest (*p* < 0.01). The contents of DE, ME, and the ratio of ME:DE were 16.46 MJ/kg DM (ranged from 13.82 to 19.13 MJ/kg DM), 15.73 MJ/kg DM (12.79 to 18.77 MJ/kg DM), and 95.44% (92.44% to 98.68%), respectively.

### 3.3. Correlation and Prediction Equations for DE and ME

As shown in Table 10, the ADF was significantly positively correlated with CF content (r = 0.91; *p* < 0.01). The GE was significantly positively correlated with CP (r = 0.63; *p* < 0.05) and NDF (r = 0.64; *p* < 0.05) contents, but negatively correlated with ash (r = −0.87; *p* < 0.01). The NDF was significantly negatively correlated with ash (r = −0.76; *p* < 0.05). The ash was significantly negatively correlated with DE (r = −0.75; *p* < 0.05) and ME (r = −0.76; *p* < 0.05), and the GE was significantly positively correlated with DE (r = 0.71; *p* < 0.05) and ME (r = 0.75; *p* < 0.05). The NDF was positively correlated with DE (r = 0.70; *p* < 0.05) and ME (r = 0.76; *p* < 0.05).

Regression equations for predicting the DE and ME of ESBM, derived from its chemical composition, are presented in Table 11. The best-fit models were as follows: DE (MJ/kg DM) = −26.31 + (2.74 × GE) − (0.17 × CP) (R^2^ = 0.76, *p* < 0.01) and ME (MJ/kg DM) = −28.45 + (2.85 × GE) − (0.19 × CP) (R^2^ = 0.70, *p* = 0.01).

### 3.4. The SID of AA in ESBM

The SIDs of AAs were significantly different among the 10 ESBM samples (*p* < 0.05) (Table 12). The mean SID of Lys, Met, Thr, Trp, Val, and TAA for ESBM samples was 81.72% (71.19 to 95.64%), 81.36% (45.59 to 95.76%), 76.19% (59.67 to 90.40%), 50.61% (15.40 to 74.13%), 81.23% (69.39 to 92.99%), and 84.29% (67.36 to 96.56%) respectively. The SID of Lys, Thr, Trp, Val, and TAA in ESBM samples 5 and 7 were the greatest (*p* < 0.01), while the SID of Lys, Thr, Trp, Val, and TAA in ESBM sample 8 were the lowest (*p* < 0.01). The SID of Met in ESBM sample 4 and 7 were the greatest, while the SID of Met in ESBM sample 8 were the lowest (*p* < 0.01). ESBM 6 and ESBM 7 showed differences in most AA.

### 3.5. Correlation Analysis and Prediction of the SID of AA in ESBM

The correlation analysis between the SID of AA and chemical composition is presented in Table 13. Ash tended to be negatively correlated with SID_Thr_, SID_Val_, and SIDTAA, while Trp showed significant positive correlations with SID_Met_, SID_Trp_, SID_Thr_, SID_Val_, and SID_TAA_, along with a positive correlation trend with SID_Lys_.

After stepwise regression analysis, the best prediction equations for the SID_Lys_, SID_Met_, SID_Trp_, SID_Thr_, SID_Val_, and SID_TAA_ in ESBM samples are presented in Table 14: SID_Lys_ = 44.80 + (61.33 × Trp) (R^2^ = 0.32, *p* = 0.09); SID_Met_ = 3.66 + (129.07 × Trp) (R^2^ = 0.51, *p* = 0.02); SID_Trp_ = (147.03 × Trp) − 37.90 (R^2^ = 0.42, *p* = 0.04); SID_Thr_ = 23.66 + (87.27 × Trp) (R^2^ = 0.51, *p* = 0.02); SID_Val_ = 74.61 + (11.04 × CF) − (6.07 × ADF) (R^2^ = 0.70, *p* = 0.02); and SID_TAA_ = 31.04 + (88.45 × Trp) (R^2^ = 0.58, *p* = 0.01).

## 4. Discussion

### 4.1. Chemical Composition

The ten types of ESBM selected for this study were sourced from prominent manufacturers across eight provinces in China, providing reasonable representativeness. ESBM is produced by hydrolyzing conventional SBM with exogenous enzymes under optimized temperature, pH, and humidity conditions [32]. This process partially degrades key ANFs in SBM such as soybean antigenic proteins, non-starch polysaccharides, and phytic acid [33,34].

This study revealed substantial variations in the chemical composition of ESBM from different sources. This variation may arise from differences in SBM sources and enzyme treatment techniques across manufacturers. Similarly, research has reported CV values exceeding 10% for CP and NDF in 11 SBM samples, with CV for minerals reaching up to 120% [35]. Enzymolysis technology can alter the chemical composition of ESBM [36]. Therefore, we recommend that prior to using ESBM in swine feed formulations, the mineral content of the specific batch should first be determined.

The mean GE of the ten ESBM samples was 17.58 MJ/kg (ranging from 16.06 to 18.76 MJ/kg). Although the maximum value approached the NRC (2012) (18.62 MJ/kg) [1], most samples fell below this level, likely due to the lower energy content of the SBM used as the substrate. The mean CP level was 49.62% (range from 40.29 to 58.6%). While some samples were consistent with the literature values (50.38%; 42.15%) [34,37], most were lower than the NRC (2012) (55.62%) [1]. Wang et al. (2022) indicated that the CP content of SBM ranges from 40% to 50% [35].

The CV for CF, NDF, and ADF were 22.24%, 16.62%, and 19.30% respectively. This is consistent with the observation that the CV for fiber in SBM is greater than 15%, highlighting the significant impact of SBM sources on the nutritional value of ESBM—especially considering that the fiber content of dehulled SBM is typically lower than that of non-dehulled SBM [35]. The ESBM used in this study had lower levels of ANFs compared to conventional SBM, which is consistent with the literature reports [38].

### 4.2. Energy Content

The test diets were formulated by substituting 30% corn with ESBM, which led to variations in dietary CP levels. However, the substitution rate was kept constant, and the basal corn diet was used to accurately correct for the energy contribution of corn. This substitution method is widely accepted and used in energy evaluation studies for single ingredients [35,39]. Therefore, the calculated DE and ME values for the ESBM samples are reliable and comparable.

Currently, there is limited data evaluating the nutritional value of ESBM from different sources for growing pigs. In this study, the DE and ME of ten ESBM samples were 16.46 MJ/kg DM (ranging from 13.82 to 19.13) and 15.73 MJ/kg DM (ranging from 12.79 to 18.77), respectively. The mean values were close to the NRC (2012) (DE = 16.38 MJ/kg DM, ME = 14.79 MJ/kg DM) [1]. This study found that DE and ME were positively correlated with the GE of ESBM and significantly negatively correlated with ash content, consistent with the literature reports [40,41]. However, contrary to the previous literature [42], this study observed a positive correlation between available energy and NDF. This discrepancy might be due to the combined effects of GE and ash masking the influence of NDF. Consequently, GE and ash were used as effective predictors to establish regression equations for DE and ME in this study. An alternative explanation relates to the enzymatic process. Specifically, the use of cellulases likely breaks down some fiber into more enzymatic substrates. Although still measured as NDF, this partially hydrolyzed fiber may contribute more energy via hindgut fermentation than the NDF in conventional soybean meal. Thus, the specific processing of the SBM could yield an NDF fraction with a higher energy content. To avoid erroneous conclusions associated with NDF, the NDF indicator was excluded from the regression equation established in this study.

Furthermore, this study determined that ESBM 8 had the lowest available energy value. ESBM 8 also exhibited the darkest brown color. There was a significant correlation between ingredient color and nutrient digestibility [43]. The drying temperature of the samples also affects the quality of feedstuffs [44]. High temperatures can induce the Maillard reaction, which darkens feed color and reduces nutrient utilization [44,45]. This provides a reasonable explanation for the low available energy value observed in ESBM 8.

### 4.3. Digestibility of Amino Acids

In this study, the IAA were referenced from published data by our research group [15]. After correcting for endogenous losses, the mean SID values for the first five limiting AAs (Lys, Met, Thr, Trp, and Val) were 81.72%, 81.36%, 76.19%, 50.61%, and 81.23%, respectively. These values were lower than those reported by NRC (2012) [1]. However, the SID of AA in ESBM 5 and ESBM 7 was significantly greater, at 94.67% and 96.56%, respectively, which was close to the reported values (93.7%) [46]. In this study, the SID of Pro exceeded 100% due to increased endogenous Pro excretion induced by the nitrogen-free diet method [47]. Furthermore, ESBM 8 exhibited the lowest SID of AA, consistent with the results of energy value evaluation. This further confirms that ESBM 8 has a poor energy value and AA digestibility, indicating its overall lower quality. As mentioned earlier, the potential reasons may be related to the source of SBM and the enzymatic hydrolysis process. Due to the low digestibility of some ingredients, the average SID of Trp was only 50.61% (15.40% to 74.13%). However, the SID of Trp for ESBM 5 and 7 was 74.13% and 71.95%, respectively. Trp is the most labile amino acid and is highly susceptible to degradation during processing, particularly under excessive heat, alkaline conditions, or in the presence of oxidizing agents. This study found that ESBM 7 had higher SID AA than ESBM 6 from the same manufacturer, suggesting that the production process affects AA digestibility.

During the development of the stepwise regression model, the first five limiting amino acids (Lys, Met, Thr, Trp, and Val) were initially included as potential predictors. However, only Trp content remained as a statistically significant variable in the final model. This study found a significant negative correlation between SID of AA and the ash content of ESBM, which was consistent with the trends observed in energy value assessments. The prediction models for the SID of AA were characterized by modest R^2^ values, implying that the variation in AA digestibility is not fully captured by the chemical composition data. While numerous studies have evaluated the nutritional value of various feedstuffs and established prediction equations for energy values, few provide equations for SID of AA [15,48]. Even when such equations are available in the literature, their R^2^ values remain low [49,50]. This suggests that further analysis of the relationship between the molecular structure of protein in feedstuffs and SID of AA is necessary to improve the prediction accuracy of SID of AA. Consequently, while these equations provide a preliminary tool for estimating the SID of AA based on chemical analysis, their predictive precision is limited. Future research should focus on incorporating more refined physicochemical properties to develop more accurate prediction models.

## 5. Conclusions

In conclusion, the chemical composition, energy content, and AA digestibility of ESBM in China have great variation. ESBM5 had the highest energy content and amino acid digestibility, while ESBM8 had the lowest nutritional value. DE and ME can be moderately predicted using GE and ash. The SID of AA can be predicted using Trp content. Additionally, more data are required to improve the accuracy of the prediction equation. These findings enable the precise application of ESBM in growing pig diet formulation, highlighting that selecting high-quality ESBM based on its chemical composition can enhance economic returns.

## Figures and Tables

**Table 1:** Source of enzymolytic soybean meal (ESBM) samples.

Number ^1^	Manufacturers ^2^	Location
ESBM 1	A	Sichuan
ESBM 2	B	Guangdong
ESBM 3	C	Shanghai
ESBM 4	D	Liaoning
ESBM 5	E	Guangdong
ESBM 6	F	Jiangsu
ESBM 7	F	Jiangsu
ESBM 8	G	Guangdong
ESBM 9	H	Hebei
ESBM 10	H	Hebei

^1^ ESBM, enzymolytic soybean meal. ^2^ Those with the same capital letter were manufactured by the same company.

**Table 2:** Formulation of diets in Experiment 1 (as-fed basis, %).

Item	Corn-Basal Diet	ESBM Diets (*n* = 10) ^1^
Corn	97.00	67.90
Enzymolytic soybean meal	-	29.10
Dicalcium phosphate	1.20	1.20
Limestone	1.00	1.00
Sodium chloride	0.30	0.30
Vitamin–mineral premix ^2^	0.50	0.50
Total	100.00	100.00

^1^ The ten enzymolytic soybean meal (ESBM) samples were included in the diets at an identical substitution ratio. ^2^ The vitamin–mineral premix provided the following per kilogram of complete diet: 1500 IU of vitamin A; 200 IU of vitamin D_3_; 10 IU of vitamin E; 0.5 mg of vitamin K_3_; 1.0 mg of vitamin B_1_; 2.5 mg of riboflavin; 1.0 mg of vitamin B_6_; 10.0 μg of vitamin B_12_; 10.0 mg of niacin; 10.0 mg of pantothenic acid; 0.3 mg of folic acid; 0.05 mg of biotin; 0.50 g of choline; 80 mg of iron (as ferrous sulfate); 10 mg of copper (as copper sulfate); 80 mg of zinc (as zinc sulfate); 30 mg of manganese (as manganese sulfate); 0.3 mg of iodine (as potassium iodide); and 0.30 mg of selenium (as sodium selenite).

**Table 3:** Analyzed nutrient content of diets in Experiment 1 (as-fed basis, %).

Item	DM ^1^	GE (MJ/kg)	CP	Ash	EE	Ca	P
Corn-basal diet	86.94	14.96	7.35	3.41	3.04	0.73	0.43
ESBM ^2^ diet							
1	89.61	15.78	21.65	5.45	2.31	0.83	0.60
2	88.82	15.70	20.35	5.03	2.27	0.83	0.57
3	88.88	15.85	19.15	5.04	2.32	0.88	0.55
4	88.16	15.92	19.62	4.98	2.33	0.88	0.57
5	89.01	16.43	21.58	4.16	2.14	0.85	0.53
6	89.15	15.96	19.30	5.19	2.34	0.86	0.56
7	89.22	15.85	19.21	4.70	2.86	0.83	0.54
8	88.98	15.54	20.41	5.69	2.44	0.90	0.58
9	87.73	15.42	16.91	5.55	2.18	0.84	0.53
10	87.55	15.34	17.44	5.10	2.21	0.81	0.52

^1^ DM, dry matter; GE, gross energy; CP, crude protein; EE, ether extract; Ca, calcium; P, phosphorus. ^2^ ESBM, enzymolytic soybean meal, sources are described in Table 1.

**Table 4:** Formulation of diets in Experiment 2 (as-fed basis, %).

Item	ESBM Diet (*n* = 10) ^2^
Enzymolytic soybean meal	40.00
Cornstarch	44.45
Sucrose	10.00
Soybean oil	3.00
Dicalcium phosphate	1.10
Limestone	0.70
Sodium chloride	0.30
Titanium dioxide	0.20
Vitamin–mineral premix ^1^	0.25
Total	100.00

^1^ Premix provided per kg of complete diet is the same as that in Table 2. ^2^ The ten enzymolytic soybean meal (ESBM) samples were included in the diets at an identical substitution ratio.

**Table 5:** Amino acid composition of experimental diets in Experiment 2 (as-fed basis, %).

Items	ESBM Diet Number ^1^									
	1	2	3	4	5	6	7	8	9	10
Dry matter	92.14	91.15	91.73	90.42	91.03	91.58	91.85	91.3	91.1	89.63
Essential AA										
Lys	1.32	1.08	0.98	1.05	1.44	1.08	1.25	1.19	0.97	1.06
Met	0.22	0.16	0.17	0.16	0.20	0.16	0.16	0.23	0.16	0.13
Thr	0.85	0.81	0.76	0.74	0.93	0.75	0.78	0.79	0.64	0.67
Trp	0.12	0.10	0.09	0.09	0.12	0.11	0.10	0.09	0.09	0.10
Val	1.05	0.98	0.92	0.92	1.13	0.89	0.92	1.13	0.80	0.83
Ile	0.96	0.91	0.85	0.87	1.05	0.85	0.87	1.05	0.74	0.77
Leu	1.73	1.61	1.49	1.50	1.84	1.49	1.52	1.71	1.30	1.36
Arg	1.64	1.36	1.24	1.24	1.51	1.24	1.31	0.58	1.10	1.18
His	0.55	0.50	0.47	0.48	0.58	0.49	0.50	0.50	0.41	0.43
Phe	1.23	1.08	1.02	0.98	1.19	1.00	1.03	0.94	0.89	0.96
Nonessential AA										
Ala	1.01	0.97	0.88	0.90	1.05	0.85	0.87	1.18	0.77	0.78
Asp	2.43	2.32	2.19	2.17	2.54	2.17	2.21	2.47	1.84	1.86
Cys	0.15	0.20	0.21	0.17	0.24	0.10	0.17	0.27	0.10	0.16
Glu	4.29	3.75	3.58	3.41	3.93	3.64	3.58	3.86	3.04	3.11
Gly	0.95	0.98	0.82	0.83	1.00	0.81	0.84	0.97	0.73	0.75
Pro	1.00	0.98	0.86	0.89	1.05	0.84	0.82	0.94	0.74	0.77
Ser	1.06	1.04	0.96	0.93	1.17	0.95	1.00	1.02	0.83	0.86
Tyr	0.72	0.64	0.67	0.61	0.74	0.64	0.67	0.69	0.56	0.67
Total AA	21.28	19.46	18.16	17.96	21.70	18.08	18.59	19.61	15.71	16.46

^1^ ESBM, enzymolytic soybean meal, sources of ESBM in each ESBM number diet are the same in Table 1.

**Table 6:** Analyzed nutrient composition of enzymolytic soybean meal (ESBM) samples (as-fed basis, %) ^1^.

Item	ESBM Number ^1^										Mean	CV
	1	2	3	4	5	6	7	8	9	10		
DM ^2^	95.82	92.79	93.53	90.53	93.13	93.62	94.45	93.13	89.48	88.32	92.48	2.38
GE(MJ/kg)	18.16	18.07	17.74	17.54	18.76	17.79	18.20	17.27	16.06	16.22	17.58	4.65
CP	58.60	52.21	49.01	48.30	55.78	48.19	47.73	52.93	40.29	43.12	49.62	10.54
EE	0.56	0.70	0.84	1.30	0.81	0.92	3.05	0.70	0.50	0.21	0.96	77.97
CF	4.42	4.60	6.09	5.84	8.87	4.70	5.13	5.20	7.07	5.80	5.77	22.24
NDF	10.07	14.34	12.97	12.97	17.72	14.94	11.98	11.37	12.33	10.62	12.93	16.62
ADF	8.83	7.21	10.72	9.98	13.67	7.66	7.34	9.67	10.33	9.52	9.49	19.30
Ash	9.26	6.42	6.63	6.59	3.89	7.63	6.14	8.54	8.66	7.46	7.12	20.71
Starch	1.40	2.34	2.04	1.78	1.90	3.91	6.63	0.82	10.24	6.28	3.73	77.23
IDF	5.85	3.95	4.30	3.55	5.25	11.40	4.10	2.95	7.20	3.70	5.23	45.48
SDF	18.45	17.95	17.45	18.60	29.45	10.50	18.05	8.80	16.60	17.15	17.30	30.07
TDF	24.30	21.90	21.75	22.15	34.70	21.90	22.15	11.75	23.80	20.85	22.53	23.22
Essential amino acid												
Lys	3.42	2.74	2.43	2.70	3.59	2.72	2.98	2.80	2.35	2.69	2.84	13.13
Met	0.56	0.40	0.33	0.39	0.39	0.35	0.27	0.44	0.32	0.29	0.37	21.22
Thr	2.16	2.01	1.79	1.86	2.24	1.75	1.77	1.77	1.45	1.63	1.84	12.22
Trp	0.55	0.57	0.62	0.63	0.61	0.47	0.64	0.42	0.51	0.56	0.56	12.59
Val	2.73	2.45	2.13	2.29	2.74	2.19	2.23	2.58	1.91	2.07	2.33	11.49
Ile	2.53	2.33	2.07	2.19	2.57	2.13	2.06	2.47	1.79	1.99	2.21	10.92
Leu	4.33	3.98	3.54	3.74	4.43	3.64	3.55	3.92	3.11	3.33	3.76	10.56
Arg	4.30	3.50	2.95	3.12	3.70	3.06	3.07	2.90	2.58	2.96	3.21	14.59
His	1.40	1.26	1.12	1.20	1.40	1.17	1.16	1.13	0.93	1.05	1.18	11.66
Nonessential amino acid												
Phe	3.05	2.64	2.39	2.44	2.91	2.61	2.37	2.07	2.22	2.22	2.49	11.85
Ala	2.57	2.35	1.95	2.19	2.51	1.99	1.99	2.56	1.70	1.94	2.17	13.36
Asp	6.26	5.80	5.23	5.36	5.97	5.06	4.98	5.61	4.17	4.87	5.33	10.85
Cys	0.43	0.57	0.43	0.45	0.63	0.24	0.48	0.72	0.25	0.41	0.46	30.91
Glu	10.95	9.36	8.37	8.70	9.52	8.53	8.10	8.58	6.91	7.72	8.67	12.00
Gly	2.41	2.41	1.89	2.05	2.41	1.91	1.93	2.18	1.68	1.82	2.07	12.36
Pro	2.39	2.39	2.18	2.22	2.47	2.13	2.11	2.14	1.82	1.90	2.18	9.07
Ser	2.65	2.53	2.23	2.36	2.84	2.19	2.21	2.26	1.86	2.07	2.32	11.72
Tyr	1.94	1.74	1.60	1.61	1.94	1.88	1.58	1.63	1.58	1.50	1.70	9.12
Total amino acid	54.62	49.02	43.27	45.49	52.86	44.01	43.49	46.15	37.13	41.00	45.70	10.97

^1^ The sources of the enzymolytic soybean meal (ESBM) samples are provided in Table 1. ^2^ DM, dry matter; GE, gross energy; CP, crude protein; EE, ether extract; CF, crude fiber; NDF, neutral detergent fiber; ADF, acid detergent fiber; SDF, soluble dietary fiber; IDF, insoluble dietary fiber; TDF, total dietary fiber; CV, coefficient of variation.

**Table 7:** Mineral composition of the enzymolytic soybean meal (ESBM) samples (as-fed basis).

Number ^1^	Ca (%)	P (%)	Cu (mg/kg)	Zn (mg/kg)	Fe (mg/kg)	Mn (mg/kg)	Na (%)	Mg (%)	K (%)
1	0.31	0.75	8.22	38.92	226.36	45.03	1.48	0.36	1.97
2	0.32	0.67	11.90	53.97	280.93	40.08	0.04	0.32	2.22
3	0.37	0.63	13.10	50.18	262.05	37.27	0.05	0.31	2.13
4	0.34	0.64	11.32	48.20	208.14	114.63	0.10	0.35	2.15
5	0.37	0.54	9.94	57.15	212.60	42.68	0.04	0.19	0.99
6	0.30	0.61	11.12	45.21	335.90	31.78	0.62	0.35	2.08
7	0.31	0.60	12.84	47.17	400.96	38.54	0.07	0.27	2.05
8	0.47	0.72	11.33	53.82	256.53	35.63	0.01	0.50	2.43
9	0.27	0.51	10.46	40.90	198.56	26.31	1.60	0.27	1.78
10	0.27	0.54	10.32	40.52	231.85	27.30	0.99	0.28	1.87
Mean	0.33	0.62	11.06	47.60	261.39	43.93	0.50	0.32	1.97
CV ^2^	17.24	12.19	12.28	12.50	23.18	55.27	120.60	24.24	18.78

^1^ The sources of the enzymolytic soybean meal (ESBM) samples are provided in Table 1. ^2^ CV, coefficient of variation.

**Table 8:** Anti-nutritional factors in enzymolytic soybean meal samples (as-fed basis).

Number ^1^	Phytic Acid (%)	Glycinin (mg/g)	β-Conglycinin (mg/g)	Trypsin Inhibitor (mg/g)	Lectin (mg/g)
1	0.25	49.19	19.85	16.17	8.83
2	0.18	108.48	122.70	11.48	9.65
3	0.19	78.59	20.26	14.87	8.77
4	0.02	18.52	1.21	14.37	9.13
5	0.14	159.30	163.04	15.57	10.87
6	0.14	40.59	2.59	17.05	8.45
7	0.27	173.50	140.45	16.66	12.90
8	0.26	16.11	1.65	14.27	9.42
9	0.25	53.83	27.26	7.00	10.71
10	0.25	28.97	0.99	6.74	9.23
Mean	0.20	72.71	50.00	13.42	9.80
CV ^2^	38.32	74.02	123.16	26.79	13.05

^1^ The sources of the enzymolytic soybean meal (ESBM) samples are provided in Table 1. ^2^ CV, coefficient of variation.

**Table 9:** DE and ME in diets and ingredients for growing pigs (MJ/kg) ^1^.

Item	ESBM Number ^1^										Mean	SEM	*p*-Value
	1	2	3	4	5	6	7	8	9	10			
Diets													
As-fed basis													
DE ^2^	14.16 ^bcde^	14.26 ^bcd^	14.18 ^bcde^	14.68 ^ab^	15.02 ^a^	14.47 ^bc^	14.42 ^bcd^	13.53 ^f^	13.95 ^def^	13.74 ^ef^	14.24	0.11	<0.01
ME	13.78 ^bcd^	13.82 ^bcd^	13.72 ^cd^	14.35 ^ab^	14.75 ^a^	13.98 ^bcd^	14.07 ^bcd^	13.08 ^e^	13.60 ^cde^	13.52 ^cde^	13.87	0.12	<0.01
Dry-matter basis													
DE	14.78 ^ef^	15.37 ^cd^	15.16 ^cde^	16.22 ^a^	16.13 ^ab^	15.46 ^cd^	15.00 ^def^	14.53 ^f^	15.59 ^bc^	15.56 ^bcd^	15.38	0.11	<0.01
ME	14.38 ^ef^	14.90 ^cde^	14.66 ^de^	15.85 ^a^	15.84 ^ab^	14.94 ^cde^	14.63 ^def^	14.04 ^f^	15.20 ^cde^	15.31 ^abc^	14.98	0.13	<0.01
ME/DE, %	97.32	96.93	96.76	97.76	98.22	96.63	97.56	96.62	97.51	98.38	97.37	0.47	0.11
Ingredients													
As-fed basis													
DE	14.96 ^bcd^	15.29 ^bc^	15.01 ^bcd^	16.69 ^ab^	17.81 ^a^	15.99 ^b^	15.82 ^bc^	12.87 ^e^	14.26 ^cde^	13.55 ^de^	15.22	0.35	<0.01
ME	14.25 ^bc^	14.39 ^bc^	14.03 ^c^	16.15 ^ab^	17.48 ^a^	14.93 ^bc^	15.21 ^bc^	11.91 ^d^	13.66 ^cd^	13.37 ^cd^	14.54	0.40	<0.01
Dry-matter basis													
DE	15.61 ^cd^	16.48 ^c^	16.04 ^c^	18.43 ^ab^	19.13 ^a^	17.08 ^bc^	16.75 ^bc^	13.82 ^d^	15.94 ^c^	15.34 ^cd^	16.46	0.38	<0.01
ME	14.88 ^cd^	15.51 ^c^	15.01 ^c^	17.84 ^ab^	18.77 ^a^	15.94 ^bc^	16.10 ^bc^	12.79 ^d^	15.27 ^c^	15.14 ^c^	15.73	0.43	<0.01
ME/DE, %	95.30	94.14	93.53	96.81	98.16	93.38	96.15	92.44	95.84	98.68	95.44	1.54	0.11

^1^ The sources of the enzymolytic soybean meal (ESBM) samples are provided in Table 1. ^2^ DE, digestible energy; ME, metabolizable energy; SEM, standard error of the mean. ^a–f^ Means in the same column with different superscript letters are significantly different at *p* < 0.05.

**Table 10:** Correlations between chemical composition and energy content in enzymolytic soybean meal (n = 10) (dry-matter basis).

Item ^1^	DE	ME	CP	EE	CF	NDF	ADF	Ash	GE
DE	1.00								
ME	0.98 **	1.00							
CP	0.11	0.14	1.00						
EE	0.26	0.29	−0.13	1.00					
CF	0.55	0.48	−0.12	−0.17	1.00				
NDF	0.70 *	0.76 *	0.13	−0.02	0.58	1.00			
ADF	0.41	0.34	0.11	−0.36	0.91 **	0.43	1.00		
Ash	−0.75 *	−0.76 *	−0.28	−0.38	−0.48	−0.76 *	−0.36	1.00	
GE	0.71 *	0.75 *	0.63 *	0.34	0.17	0.64 *	0.15	−0.87 **	1.00

^1^ DE, digestibility energy; ME, metabolizable energy; CP, crude protein; EE, ether extract; CF, crude fiber; NDF, neutral detergent fiber; ADF, acid detergent fiber; GE, gross energy; * 0.01 ≤ *p* < 0.05, ** *p* < 0.01.

**Table 11:** Prediction equations for DE and ME of enzymolytic soybean meal derived from chemical composition (dry-matter basis).

Item ^1^	Prediction Equations ^2^	R^2^	RMSE	*p*-Value
1	DE(MJ/kg DM) = −26.31 + (2.74 × GE) − (0.17 × CP)	0.76	0.85	<0.01
2	DE(MJ/kg DM) = 21.71 − (0.68 × Ash)	0.57	1.06	0.01
3	ME(MJ/kg DM) = −28.45 + (2.85 × GE) − (0.19 × CP)	0.70	1.02	0.01
4	ME(MJ/kg DM) = 21.33 − (0.73 × Ash)	0.57	1.16	0.01

^1^ DE, digestible energy; ME, metabolizable energy; DM, dry matter; GE, gross energy; CP, crude protein; RMSE, root mean square error is a measure of precision. ^2^ Prediction equations were developed based on stepwise regression analyses.

**Table 12:** Standardized ileal digestibility of amino acid in enzymolytic soybean meals samples (%).

Item	Enzymolytic Soybean Meal ^1^										Mean	SEM	*p*-Value	IAA ^2^ g/kg
	1	2	3	4	5	6	7	8	9	10				g/kg DMI
Essential AA														
Lys	76.43 ^cd^	78.40 ^bcd^	72.11 ^d^	81.80 ^bc^	92.12 ^a^	73.87 ^cd^	95.64 ^a^	71.19 ^d^	88.03 ^ab^	87.61 ^ab^	81.72	2.11	<0.01	5.09
Met	72.75 ^b^	86.54 ^ab^	79.12 ^ab^	87.75 ^ab^	95.02 ^a^	77.48 ^ab^	87.57 ^ab^	45.59 ^c^	95.76 ^a^	85.99 ^ab^	81.36	4.26	<0.01	0.60
Thr	67.43 ^de^	79.53 ^abcd^	69.31 ^cde^	79.67 ^abc^	88.68 ^ab^	68.71 ^de^	90.40 ^a^	59.67 ^e^	82.23 ^ab^	76.29 ^bcd^	76.19	2.63	<0.01	6.19
Trp	42.30 ^cd^	52.10 ^bcd^	33.91 ^de^	52.77 ^bcd^	74.13 ^a^	40.13 ^cde^	71.95 ^ab^	15.40 ^e^	65.01 ^ab^	58.40 ^abc^	50.61	4.66	<0.01	0.63
Val	72.20 ^d^	83.92 ^abc^	76.22 ^cd^	84.42 ^abc^	90.79 ^ab^	70.45 ^d^	92.99 ^a^	69.39 ^d^	88.23 ^ab^	83.65 ^bc^	81.23	1.83	<0.01	5.09
Phe	79.16 ^c^	87.63 ^abc^	80.44 ^bc^	88.20 ^ab^	92.26 ^a^	81.20 ^bc^	94.55 ^a^	67.25 ^d^	90.23 ^a^	87.93 ^abc^	84.89	1.89	<0.01	3.48
Ile	74.27 ^de^	86.68 ^ab^	78.29 ^cd^	87.19 ^ab^	92.34 ^ab^	73.65 ^de^	94.56 ^a^	70.50 ^e^	88.89 ^ab^	84.74 ^bc^	83.11	1.71	<0.01	4.36
Leu	75.94 ^d^	86.38 ^abc^	78.76 ^cd^	86.97 ^ab^	91.49 ^ab^	73.33 ^d^	93.63 ^a^	71.74 ^d^	89.39 ^ab^	85.49 ^bc^	83.31	1.61	<0.01	6.38
Arg	89.30 ^c^	95.65 ^abc^	91.60 ^bc^	96.54 ^abc^	99.88 ^ab^	89.37 ^c^	100.80 ^a^	68.38 ^d^	97.50 ^abc^	96.71 ^abc^	92.57	1.83	<0.01	7.49
His	75.68 ^d^	86.92 ^abc^	79.14 ^bcd^	86.56 ^abc^	93.26 ^a^	77.11 ^cd^	95.61 ^a^	64.27 ^e^	88.61 ^ab^	85.12 ^abcd^	83.23	2.14	<0.01	2.19
Nonessential AA														
Ala	69.46 ^e^	78.91 ^bcde^	73.90 ^cde^	82.17 ^abc^	89.40 ^ab^	68.49 ^e^	92.75 ^a^	70.44 ^de^	86.59 ^ab^	80.60 ^bcd^	79.27	2.32	<0.01	8.23
Asp	62.56 ^cd^	82.84 ^ab^	71.88 ^bc^	79.70 ^ab^	91.25 ^a^	62.20 ^cd^	91.91 ^a^	53.27 ^d^	81.17 ^ab^	79.24 ^ab^	75.60	3.24	<0.01	9.69
Cys	57.36 ^ef^	79.42 ^abcd^	69.98 ^cde^	73.32 ^bcde^	89.54 ^ab^	47.48 ^f^	92.03 ^a^	64.28 ^def^	86.24 ^abc^	82.21 ^abc^	74.19	3.78	<0.01	1.20
Glu	72.24 ^de^	83.55 ^abcd^	73.75 ^cde^	79.25 ^bcd^	90.76 ^ab^	75.96 ^cde^	94.11 ^a^	63.54 ^e^	84.81 ^abc^	80.22 ^bcd^	79.82	2.65	<0.01	12.90
Gly	73.58 ^bcd^	79.89 ^abcd^	73.84 ^bcd^	86.55 ^abc^	93.25 ^a^	68.20 ^cd^	98.71 ^a^	64.75 ^d^	92.45 ^ab^	84.12 ^abcd^	81.53	4.20	<0.01	21.04
Pro	146.51	140.12	143.16	155.95	146.14	114.86	165.99	119.87	163.27	163.93	145.98	12.59	0.08	84.34
Ser	74.12 ^de^	86.97 ^abc^	77.41 ^cd^	84.87 ^abc^	92.84 ^ab^	73.89 ^de^	94.10 ^a^	67.68 ^e^	84.57 ^abc^	83.01 ^bcd^	81.95	2.09	<0.01	6.65
Tyr	79.47 ^c^	85.85 ^bc^	80.02 ^c^	88.03 ^ab^	94.17 ^a^	78.11 ^c^	94.03 ^a^	67.54 ^d^	90.55 ^ab^	89.12 ^ab^	84.69	1.57	<0.01	2.95
TAA	76.90 ^bcd^	87.06 ^abcd^	79.25 ^bcd^	87.41 ^abc^	94.67 ^a^	75.19 ^cd^	96.56 ^a^	67.36 ^d^	90.91 ^ab^	87.57 ^abcd^	84.29	2.37	<0.01	192.18

^1^ The sources of the enzymolytic soybean meal (ESBM) samples are provided in Table 1. ^2^ The IAA values (g/kg DMI) used for the calculation of SID were derived from a previous study conducted by our group [15]. ^a–f^ Means in the same column with different superscript letters are significantly different at *p* < 0.05.

**Table 13:** Correlation coefficients between chemical composition and standardized ileal digestibility of amino acid in the 10 enzymolytic soybean meals for growing pigs (as-DM basis).

Item	SID_Lys_	SID_Met_	SID_Trp_	SID_Thr_	SID_Val_	SID_TAA_
Ash	−0.43	−0.45	−0.50	−0.63 *	−0.55 *	−0.56 *
Trp	0.57 *	0.71 **	0.65 **	0.71 **	0.72 **	0.76 **

TAA, total amino acids; * 0.05 ≤ *p* < 0.10, ** 0.01 ≤ *p* < 0.05.

**Table 14:** Stepwise regression equation for standardized ileal digestibility of amino acids based on the chemical composition of the 10 enzymolytic soybean meals for growing pigs (as-DM basis).

Item	Linear Regression Equations	R^2^	RMSE	*p*-Value
1	SID_Lys_ = 44.80 + (61.33 × Trp)	0.32	7.61	0.09
2	SID_Met_ = 3.66 + (129.07 × Trp)	0.51	10.80	0.02
3	SID_Trp_ = (147.03 × Trp) − 37.90	0.42	14.72	0.04
4	SID_Thr_ = 23.66 + (87.27 × Trp)	0.51	7.34	0.02
5	SID_Thr_ = 104.31 − (3.65 × Ash)	0.39	8.13	0.06
6	SID_Val_ = 34.83 + (77.07 × Trp)	0.52	6.34	0.02
7	SID_Val_ = 74.61 + (11.04 × CF) − (6.07 × ADF)	0.70	5.37	0.02
8	SID_TAA_ = 31.04 + (88.45 × Trp)	0.58	6.40	0.01
9	SID_TAA_ = 108.17 − (3.10 × Ash)	0.32	8.16	0.09

CF, crude fiber; ADF, acid detergent fiber; TAA, total amino acids; RMSE, root mean square error.

## Data Availability

The original contributions presented in the study are included in the article. Further inquiries can be directed to the corresponding authors.
